# Genomic Profiling of Patient-Derived Colon Cancer Xenograft Models

**DOI:** 10.1097/MD.0000000000000298

**Published:** 2014-12-02

**Authors:** Won-Suk Lee, Hye-Youn Kim, Jae Yeon Seok, Ho Hee Jang, Yeon Ho Park, So-Young Kim, Dong Bok Shin, Suntaek Hong

**Affiliations:** From the Department of Surgery, Gil Medical Center, Gachon University (WSL, YHP, SYK); Lee Gil Ya Cancer and Diabetes Institute, Gachon University (WSL, HYK, HHJ, SYK, SH); Department of Hemato-Oncology, Gil Medical Center, Gachon University (DBS); Department of Pathology, Gil Medical Center, Gachon University (JYS); and Gachon Medical Research Institute, Gil Medical Center, Incheon, Korea (HHJ, SH).

## Abstract

Recent evidence suggests that patient derived xenograft (PDX) models can maintain certain pathological and molecular features of the original disease. However, these characterizations are limited to immunohistochemistry or by tissue microarray analysis. We conducted a high-throughput sequencing of primary colon tumor and PDX has not been reported yet.

Fresh primary colon cancer tissues that originate from surgery were implanted into the subcutaneous space of 6- to 8-week-old female BALB/c nu/nu or NOD/SCID mice and serially passaged in vivo. Ion AmpliSeq Cancer Hotspot Panel v2 (Ion Torrent) was used to detect frequent somatic mutations and similarity of molecular characteristics between the 10 patient tumors and matched PDX.

Histologic and immunohistochemical analyses revealed a high degree of pathologic similarity including histologic architecture and expression of CEA, CK7, and CD20 between the patient and xenograft tumors. In 80% cases, all of the somatic mutations detected in primary tumor were concordantly detected in PDX models. However, 2 PDX models showed gained mutations such as PIK3CA or FBWX7 mutation.

Ten patient-derived advanced colon cancer xenograft models were established. These models maintained the key characteristic features of the original tumors, suggesting useful tool for preclinical personalized medicine platform.

## INTRODUCTION

While colorectal cancer (CRC) is the third most common cancer in the world,^1^ with nearly 1.4 million new cases diagnosed in 2012. Despite improvements in the systemic therapy of CRC over the last 2 decades, almost half of all patients who undergo surgical resection with curative intent experience relapse, and there remains a pressing unmet need for more effective therapies informed by our increasing knowledge of CRC biology.^2^ Recently, several targeted therapeutics for CRC have been discovered, which provide additional options for physicians and patients.^3,4^

Preclinical evaluation of targeted therapies predominantly rely on the use of animal tumor models,^5^ and the transplantation of standard tumor cell lines into mice to generate xenografts is common practice in preclinical drug discovery.^6,7^ However, the prolonged in vitro artificial culturing causes transplanted tumor cells to no longer maintain the original molecular characteristics and show heterogeneity of the patient tumor.^8–10^ One of the most profound issues with using standard xenograft models is their poor predictive power for the translation of preclinical efficacy into clinical outcome.^11,12^

In the era of targeted therapy, mutation profiling of the cancer is becoming more influential on therapeutic decisions. Compared to standard cell-line derived xenograft models, the greatest advantage of patient-derived xenograft (PDX) models is their ability to better predict clinical tumor response. Recent evidence suggests that PDX models can maintain certain pathological and molecular features of the original disease.^13,14^ However, to the best of our knowledge, high-throughput sequencing of primary colon tumor and PDX has not been reported yet.

The application of the next generation sequencing (NGS) technology to cancer research has led to dramatic advances in the understanding of genomic background of cancers. One of the NGS platforms, the Ion Torrent AmpliSeq Cancer Panel, which relies on non-optical detection of hydrogen ions in a semiconductor device,^15^ and is able to detect 2855 oncogenic mutations in 50 commonly mutated genes (Supplementary Table 1).

The goal of this study was to create the PDX model from human advanced primary colon cancers in nude mice and to characterize how faithfully the mutational status of oncogenes of xenografts recapitulates that of the original tumors by means of Ion Torrent AmpliSeq Cancer Hotspot Panel.

## METHODS

### Patient and PDX Samples

Tumor areas (>75%) were dissected under microscopy from 4 μm unstained sections by comparison with a H&E stained slide, and genomic DNA was extracted using a Qiagen DNA FFPE Tissue Kit (Qiagen, Hilden, Germany) according to the manufacturer's instructions from 10 patients with advanced colon cancer.

### Ethics Statement

All experiments involving animals were approved in advance by Animal Ethics Committee at Lee Gil Ya Cancer and Diabetes Institute, Gachon University, Incheon, Korea and were carried out in accordance with Australian code of practice for the Care and Use of Animals for Scientific Purposes. Written informed consent was obtained from each patient and the study was approved by the Gil hospital ethics committee.

### Ion AmpliSeq Cancer Hotspot Panel v2

**FIGURE 1:**
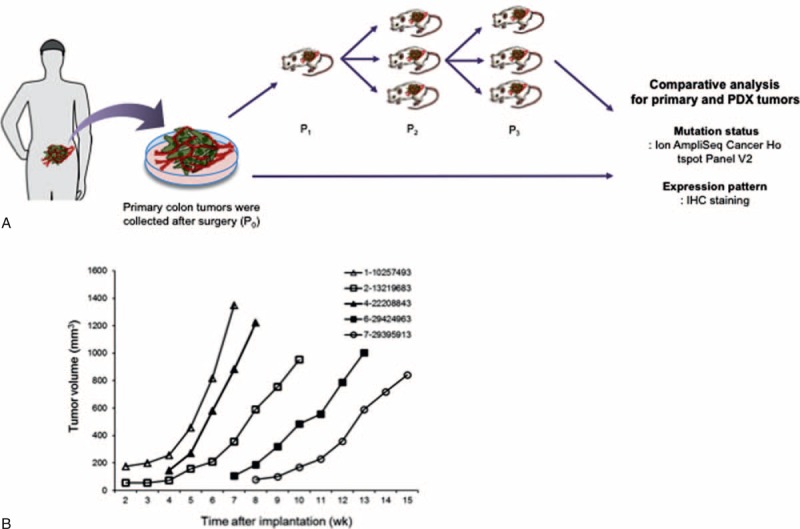
Establishment and validation of PDX model for human colorectal cancer. (A) Surgically removed tumor tissues were implanted subcutaneously into athymic nude mice. After growing up to 1000 mm^3^, tumors were harvested and immediately re-implanted for expansion. After finishing inoculation, the remaining tumor tissues were cryopreserved in liquid nitrogen tank for later use or re-implantation. Then, Ion AmpliSeq Cancer Hotspot Panel v2 was used for mutational analysis of primary and xenograft tumor to confirm the similarity between 2 samples. (B) To check the availability of established PDX models, cryopreserved tumors were implanted into mice and measured the growth curve of each tumor.

We used the Ion AmpliSeq Cancer Hotspot Panel v2 (Ion Torrent) to detect frequent somatic mutations that were selected based on literature review. It examines 2855 mutations in 50 commonly mutated oncogenes and tumor suppressor genes (Supplementary Table 1). First, 200 ng of DNA from each of samples underwent single-tube, multiplex PCR amplification using the Ion AmpliSeq Cancer Primer Pool and the Ion AmpliSeq Kit reagents (Life Technologies, Seoul, Korea)^16^. Treatment of the resulting amplicons with FuPa Reagent partially digested the primers and phosphorylated the amplicons. The phosphorylated amplicons were ligated to Ion Adapters and purified. For barcoded library preparation, we substituted barcoded adapters from the Ion Xpress™ Barcode Adapters 1-96 Kit for the non-barcoded adapter mix supplied in the Ion AmpliSeq^™^ Library Kit. The ligated DNA underwent nick-translation and amplification to complete the linkage between adapters and amplicons and to generate sufficient material for downstream template preparation. Two rounds of Agencourt AMPure XP Reagent binding at 0.6 and 1.2 bead-to-sample volume ratios removed input DNA and unincorporated primers from the amplicons. The final library molecules were 125 to 300 bp in size. We then transferred the libraries to the Ion OneTouch^™^ System for automated template preparation. Sequencing was performed on the Ion PGM™ sequencer according to the manufacturer's instructions. We used IonTorrent Software for automated data analysis. The general schema of this study is described in Figure 1A.

### Establishment of Patient-Derived Tumor Xenograft (PDX)

Six to eight week-old female BALB/c *nu*/*nu* or NOD/SCID mice (Orient Bio, Korea) were used for implantation of human colorectal tumor tissues. The fresh tumor tissue after surgery were rinsed with RPMI 1640 media containing antibiotics and placed on ice. Tumor tissues were cut into 10 mm^3^ pieces and implanted subcutaneously into mice (P_0_). Tumor size and body weight of mice were continuously measured up to 10 weeks with calipers and tumor volume was calculated using longitudinal (*L*) and transverse (*W*) tumor diameters with formula *V* = (*L* × *W*^2^)/2. After reaching the volume of 1000 mm^3^, mice were sacrificed and tumor tissues were collected (P_1_). Immediately, harvested tissues were re-implanted for expansion in later serial generations (P_2_, P_3_, P_4_, and P_5_). Xenografted tumor tissues were cryopreserved in liquid nitrogen tank for later use and long-term storage. Mice were housed under pathogen-free conditions and maintained on a 12-h light/dark cycle, with food and water supplied ad libitum. We performed genomic profiling at P3.

### Analytical Methods

Data from the IonPGM runs were processed initially using the Ion Torrent platform-specific pipeline software Torrent Suite to generate sequence reads, trim adapter sequences, filter, and remove poor signal-profile reads. Initial variant calling from the Ion AmpliSeq sequencing data was generated using Torrent Suite Software v4.0.2 with a plug-in “variant caller v4.0.6” program. In order to eliminate erroneous base calling, 2 filtering steps were used to generate final variant calling. The first filter was set at an average depth of total coverage of >1000, an each variant coverage of >6, a variant frequency of each sample >1%. The second filter step was to eliminate 12 SNPs (Supplementary Table 2) in our previous data. We excluded all synonymous changes after an automated mutation-calling algorithm was used to detect supposed mutations.

## RESULTS

### Establishment of PDX Colon Cancer Mouse Model

Clinical and pathologic characteristics are described in Table 1. None of the patients received chemotherapy or radiation therapy prior to surgery. The original patient colon cancer tissues were implanted into nude mice subcutaneously and then growing xenograft tissues were implanted into second generation nude mice models. The model succession rate was 100% (10/10) in the first generation of nude mice, and then 100% (20/20) in the second and subsequent generations (Figure 1A). To confirm the establishment of stable PDX model of colon cancers, cryopreserved xenograft tumors were re-implanted into nude mice and determined the growth curves of each model (Figure 1B). Some tumors grown fast after re-implantation, but other samples were shown a little slow growth rate.

**TABLE 1:**
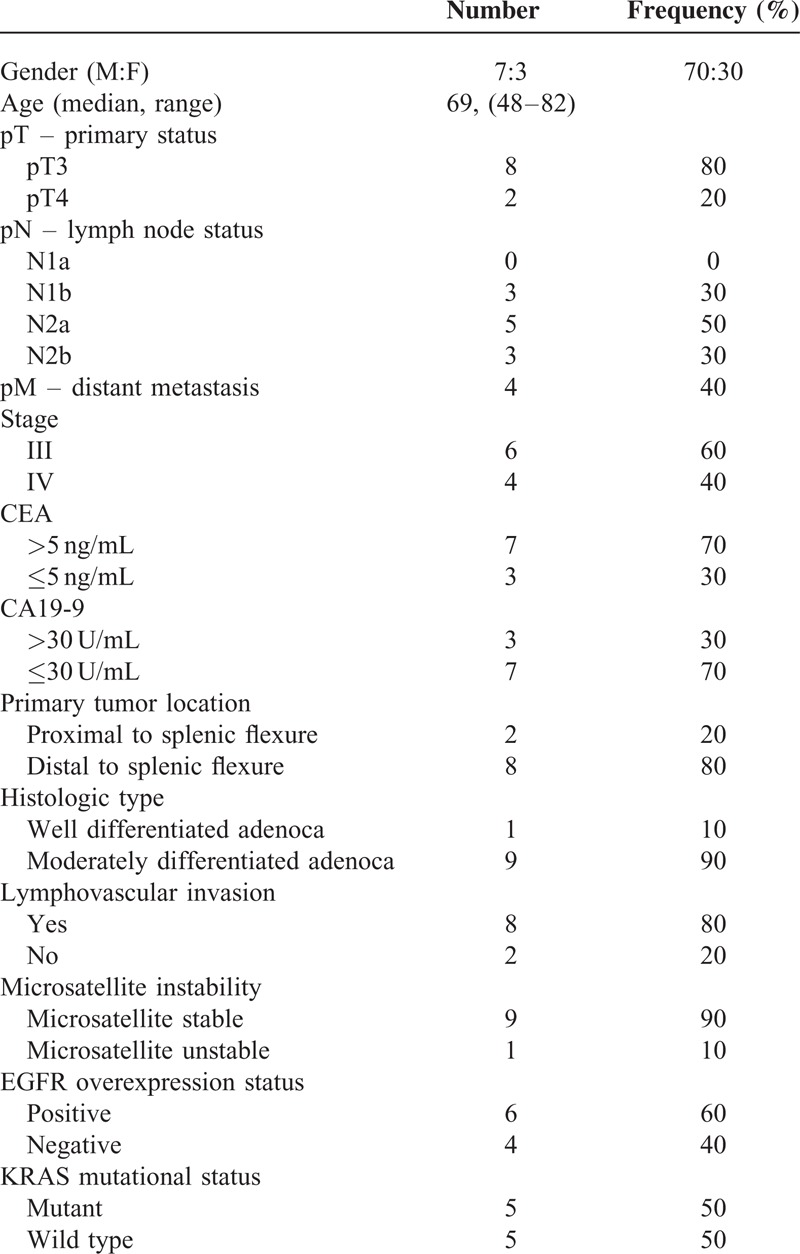
Patient Characteristics (n = 10)

**FIGURE 2:**
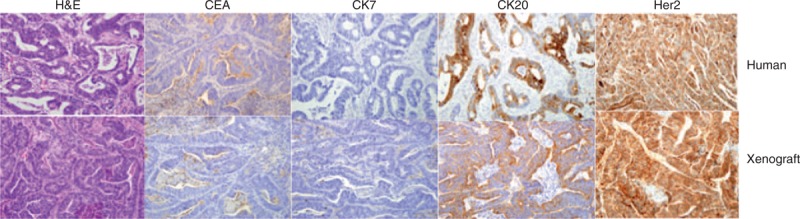
The immunohistochemical staining of primary colon tumor and their matched PDX models. A comparison of hematoxylin and eosin(H&E), CK20, CK7, and CEA stained for original tumor with xenograft tumor revealed comparable staining patterns in both original and the xenograft tumors. The tumors were positive for CK20 and negative for CK7, a pattern seen exclusively in colon cancer. One patient colon cancer (ID#22208843) and PDX showed similar patterns of HER2 overexpression.

The xenograft tissues were analyzed by hematoxylin and eosin and immunohistochemical staining for pathology assessment. The patient-derived colon cancer xenograft tissues (P_3_) exhibited similar morphology to that of the patient tissues from which the primary models were derived (Figure 2). The PDX and original tumors also showed similar patterns of expression for carcinoembryonic antigen. The tumors were positive for CK20 and negative for CK7, a pattern seen exclusively in colon cancer. One patient colon cancer (ID#22208843) and PDX showed similar patterns of HER2 overexpression (Figure 2).

### Mutational Status of Cancer-Related Genes in Primary and Xenograft Tumors

Frequently detected somatic mutations were identified in primary and PDT tumors (Table 2). Mutation profiles follow as: *TP53* (10 cases, 100%), *KRAS* (5 cases, 50%), *PIK3CA* (3 cases, 30%), *APC* (2 cases, 20%), *FBXW7* (2 cases, 20%), STK11 (2 cases, 20%), MET (2 cases, 20%), *SMARCB1* (1 cases, 10%), *ATM* (1 cases, 10%), MLH1 (1 cases, 10%), PTEN (1 cases, 10%), and ERBB2 (1 cases, 10%).

**TABLE 2:**
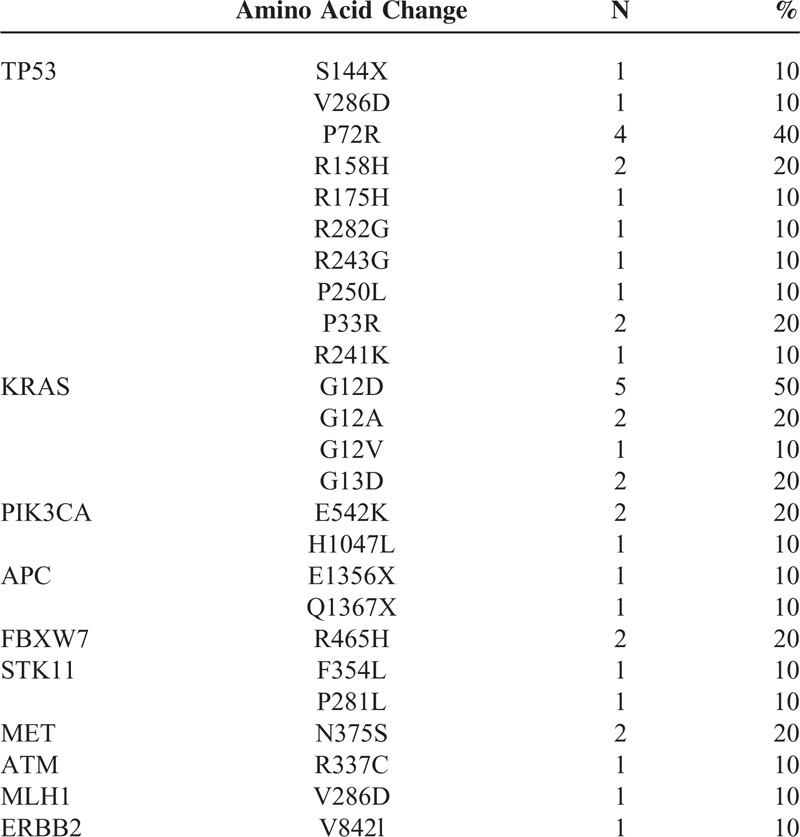
Mutation Profiles of Frequently Mutated Genes

The molecular characterization of primary tumor and PDX is described in Table 3. In 80% cases, all of the somatic mutations detected in primary tumor were concordantly detected in PDX models. The primary colon tumor (ID#10257493) harbored APC Q1367X, TP53 S144X, P33R, KRAS G12D, and MET N375S mutations. The PDX derived from the primary tumor also harbored the same mutational profile. Two PDX models were not concordant with the primary tumor (ID#30306113 and 5956103 respectively). The PDX tumor gained PIK3CA and another PDX tumor gained FBXW7, PIK3CA, PTEN (Table 3).

**TABLE 3:**
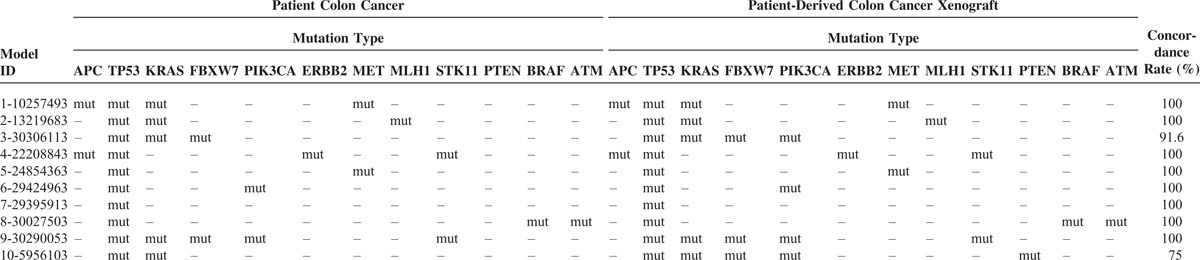
Similarity in Mutation Profiles Between Primary and PDX Tumors

**FIGURE 3:**
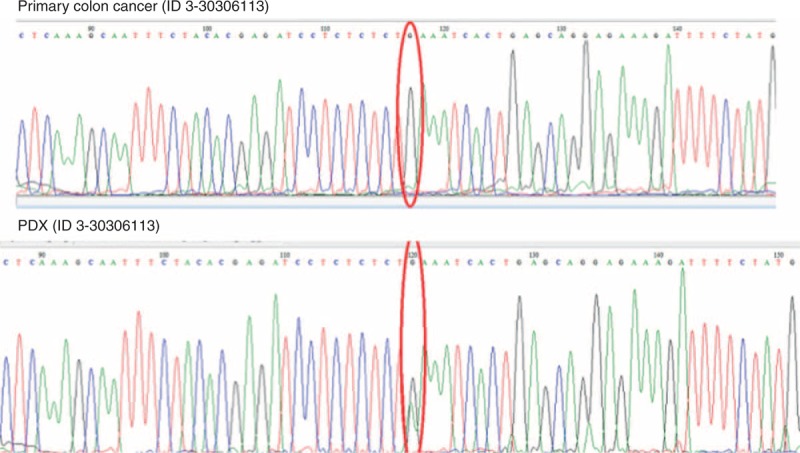
Newly emerged PIK3CA oncogenic mutation. As detected by Ion Torrent PGM, direct sequencing confirmed that PIK3CA mutation was not present but has emerged as a new oncogenic mutation with allelic frequency of 49% in xenograft model.

Of note, the primary colon cancer tissue (ID#2208843) had ERBB2 V842l mutation and PDX model had the same ERBB2 V842I mutation. Interestingly, 1 PDX model (ID#5956103) showed gained mutation in PIK3CA E542K, H1047L, and FBWX7 R465H. And another PDX model (ID#30306113) showed gained mutation in PIK3CA E542K. As detected by Ion Torrent PGM, direct sequencing confirmed that PIK3CA mutation was not present but has emerged as a new oncogenic mutation with allelic frequency of 49%. (Figure 3).

## DISCUSSION

The present work aimed to validate models of human advanced colon adenocarcinomas expanded into the nude mice in which the properties of the initial tumors are maintained or not. In this study, we characterized a group of 10 PDX with their corresponding primary tumors by using Ion Ampliseq v2. To the best of our knowledge this is the first report on comparing PDX with primary tumor using high-throughput sequencing.

One of the most important advantages in developing PDX models is that the model can better represent the genetic diversity and molecular characteristics of the original patient.^17^ We found that 9 out of 10 advanced colon adenocarcinoma samples maintained concordant somatic mutations (Tables 2 and 3). TP53 was the most frequently found mutations followed by KRAS, PIK3CA, and APC in both primary and PDX tumors.

For application of the PDX models for further molecular analysis or drug efficiency test system, established models should be validated whether PDX models represent the original human tumors. Actually, our PDX models showed same histological pattern of several markers with primary human tumors (Figure 2). Also, PDX tissues were grown well even after storage in liquid nitrogen (Figure 1B). These data suggest that our models stably established and can be used for further study. Interestingly, PDX samples containing large number of mutated genes (1-10257493 and 4-22208843) showed high growth rate compared with other tumors (6-29424963 and 7-29395913). It is reasonable that high mutation of cancer-related genes can accelerate the growth of tumors compare to another one.

A critical question regarding PDX model stability is whether through the process of engraftment and expansion changes the genetic features of the tumors. Comprehensive genome-wide gene-expression analysis studies have demonstrated that PDX maintain the majority of the key genes and global pathway activity in primary tumors.^8,18^ However, Fichtner et al reported using gene profiling (human-specific Affymetrix array) that 9 out of 17 non-small cell carcinoma PDX tumors clustered with their parent tumor using unsupervised hierarchical clustering, while of the 8 that did not. Thus, the results indicate that although there can be a high degree of concordance between primary tumor and PDX, this similarity should not be insinuated. Our data supports this finding that, we had 80% concordance rate between primary and metastasis, but 1 PDX model (ID 10-5956103) have had gained mutations such as PIK3CA and FBWX7 throughout the process. Based on our results, our PDX models can be used as a preclinical model reflective of patient's cancer in >80% of cases. However, a few cases, PDX models may harbor new mutations such as PIK3CA or FBWX7 mutations. F-box and WD40 domain protein 7 (FBWX7) is a component of E3 ubiquitin ligase, which plays an important role in mitotic checkpoint. FBWX7 is a tumor suppressor which is found mutated in various cancers (REF).^19^ Recently, FBWX7 deficient cells have shown to be a master regulator of mitotic checkpoints in cancer. Since the primary tumor did not harbor FBWX7 mutation but its corresponding PDX model harbored FBWX7 mutation, it can be speculated that FBWX7 is a genetic event developed during PDX model development. In all, this model can be tested with different targeted agents including aurora kinase, which hits the mitotic pathway deregulated by FBWX7 mutations.^19^

In addition, we found a somatic mutation in HER2 (V842l) in both primary and PDX models. In our recent study, we found that 10% of colon cancer patients had HER2 amplification and HER2-targeting agents demonstrate anti-tumor efficacy in HER2-amplified colon cancer cell lines.^20^ Recently, HER2 V842l mutation in breast cancer was shown to be activating mutations associated with drug sensitive to neratinib.^21^ We plan to test the anti-tumor efficacy of neratinib in this HER2 mutated colon cancer PDX model. In this colon cancer PDX model, both of the primary tumor and PDX model had HER2 overexpressions.

Recently, several groups have reported on the PDX establishments in colorectal cancer patients using different methods.^22,23^ One of the newly emerging techniques is to establish patient derived colon cancer cells and then establish PDX models.^23^ In line with our study, they observed that mutational profile of some of the PDX models are overly enriched when compared to the primary colon tumors. In other words, some of the major mutations are more frequently observed in PDX models while some are lost in patient derived cells before PDX enrichment. We directly established mouse PDX models with tumor samples collected from surgical specimen which is similar to the study reported by Cho et al.^22^ Although Cho et al^22^ reported that they observed 100% concordance rate in mutational profile, they only conducted hotspot mutations in BRAF, KRAS, PIK3CA, TP53, and APC. In our study, 80% of the PDX models were genomically concordant with the primary colon tumors, and 1 PDX model (ID 30306113) demonstrated a newly emerged PIK3CA oncogenic mutation (E542K) with allelic frequency of 49%. The frequency of PIK3CA E542K mutation (COSM760) in primary colon tumor (ID 30306113) did not harbor this mutation. Hence, our study suggests that high throughput sequencing may identify additional mutation in PDX models when compared with primary colon cancer.

In summary, 10 patient-derived advanced colon cancer xenograft models were established. In the era of personalized genomic medicine, these PDX models represent useful tools to further understand colon cancer and to enable development of personalized approaches for the treatment of colon cancer patients.
